# The Ethyl Acetate Extract From *Celastrus orbiculatus* Promotes Apoptosis of Gastric Cancer Cells Through Mitochondria Regulation by PHB

**DOI:** 10.3389/fphar.2021.635467

**Published:** 2021-05-28

**Authors:** Lide Tao, Zixin Yin, Tengyang Ni, Zewen Chu, Shihua Hao, Zeyu Wang, Masataka Sunagawa, Haibo Wang, Yanqing Liu

**Affiliations:** ^1^ Nanjing University of Traditional Chinese Medicine, Nanjing, China; ^2^ Department of General Surgery, Affiliated Hospital of Yangzhou University, Yangzhou University, Yangzhou, China; ^3^ Institute of Translational Medicine, Medical College, Yangzhou University, Yangzhou, China; ^4^ Dalian Medical University, Dalian, China; ^5^ Department of Physiology, School of Medicine, Showa University, Tokyo, Japan

**Keywords:** *Celastrus* orbiculatus extract, Prohibitin (PHB), gastric cancer, apoptosis, traditional Chinese medicine

## Abstract

**Objective:** To investigate the effect of ethyl acetate extract from *Celastrus orbiculatus* (COE) on gastric cancer cell apoptosis and reveal its underlying molecular mechanism. In addition, it was aimed to stablish a theoretical basis for the clinical application of *Celastrus orbiculatus* in the gastric cancer treatment.

**Material and Methods:** Western blot and RT-qPCR were used to detect mRNA and protein expression of PHB in gastric cancer and adjacent tissues. MTT method was used to detect the COE effect on the proliferation of AGS cells and to determine the 50% inhibitory concentration COE on these cells. COE effect on AGS apoptosis was evaluated by flow cytometry. Changes in apoptosis-related proteins expression in AGS cells were detected by western blot and changes in mitochondrial membrane potential were detected by JC-1 fluorescence staining. PHB expression was knocked down in AGS cells by lentiviral-mediated RNA interference. The COE antitumor effect was assessed *in vivo* using a subcutaneous transplantation tumor model in nude mice and *in vivo* fluorescence tracing technique in small animals.

**Results:** The clinical samples analysis results showed that the PHB expression in gastric cancer samples was significantly higher than in corresponding adjacent tissues. MTT results showed that the AGS cell proliferation was significantly inhibited. RT-qPCR and western blot results showed that COE can significantly inhibit the PHB mRNA and protein expression, respectively. Flow cytometry analysis showed that COE was able to significantly promote AGS cell apoptosis. Western blot results also indicated that apoptosis-related protein expression changed significantly; BCL-2 expression significantly reduced while the Caspase-3 and Bax expression significantly increased after COE treatment. JC-1 fluorescence staining results showed that COE changed the mitochondrial membrane potential and activated the mitochondrial apoptosis pathway. Furthermore, *in vivo* experiments results demonstrated that the growth of subcutaneous transplanted tumor was significantly inhibited by the PHB knockdown and by the COE intragastric administration.

**Conclusion:** COE can significantly promote apoptosis of human gastric cancer cells, which can be achieved by inhibiting PHB expression, thus altering the structure and function of mitochondria and activating the mitochondria apoptosis pathway. The antitumor effect of COE has also been proved *in vivo*.

## Background

Gastric cancer is one of the most prevalent cancers in the world. The incidence rate of gastric cancer is the first among all types of malignant tumors in China. Gastric cancer-related death ranks second among all kinds of malignant tumors worldwide (Chen et al., 2016; Bray et al., 2018). As the early symptoms of gastric cancer are not obvious, most patients are at an advanced stage in the first diagnosis, presenting lymphatic metastasis or peripheral infiltration. In addition, it is very common for metastases to occur during treatment, with high recurrence and metastasis rates even after gastric cancer resection. Gastric cancer patients generally have a poor prognosis and low sensitivity to chemotherapy due to the high metastasis rate from this type of tumor. Therefore, there is an urgent need to find new drugs to improve the survival rate of gastric cancer patients. *Celastrus orbiculatus* Thunb (Celastracea family) is a traditional medicinal plant in China with antitumor properties. Previous studies have shown that ethyl acetate extract of *Celastrus orbiculatus* (COE) can significantly inhibit epithelial mesenchymal transition (EMT), gastric cancer cell invasion and metastasis and the growth of a variety of cancer cells(Zhang et al., 2012; Zhu et al., 2014; Zhu et al., 2018). However, the inhibitory effect of COE on tumor proliferation has not been fully studied and its underlying molecular mechanism is still unclear. The elucidation the mechanism by which COE acts in antitumor cell proliferation and in apoptosis promotion can be useful for the development of new antitumor drug candidates. The mitochondrial pathway is the most common pathway of apoptosis. The function and structural stability of mitochondria directly affect the energy supply and apoptosis of tumor cells. Changes in the permeability of cell mitochondrial membranes can directly induce the release of the apoptosis-initiating factor cytochrome C, which ultimately leads to the occurrence of cell apoptosis. Our previous studies have shown that the total terpenes of *Celastrus orbiculatus* can inhibit the growth and induce apoptosis of a variety of malignant tumor cells, such as liver, gastric, colorectal and cervical cancer. The total terpenes of *Celastrus orbiculatus* can significantly inhibit growth and induce apoptosis of human gastric cancer cells in a dose-dependent manner. In addition, the total terpenes of *Celastrus orbiculatus* can inhibit the EMT induced by TGF-β1 in SGC-7901 cells, and significantly inhibit the expression levels of Cofilin 1, Heat shock protein 27 (Hsp27), Prohibitin (PHB) and Annexin A5 (Zhu et al., 2014). These data show the broad prospects for the application of *Celastrus orbiculatus* total terpenes as an antitumor agent. Based on these previous studies, the present work analyzed the effect of COE on the cell viability and apoptosis of the human gastric cancer cell line AGS. In addition, the signaling pathways that may be involved in the COE antitumor effect have been identified in order to lay the foundation for new antitumor drugs development.

## Material and Methods

### Material

#### Drugs

*Celastrus orbiculatus* Thunb. was purchased from Guangzhou Zhixin Pharmaceutical Co., Ltd. (Batch No.: 070510). Its identification as provided by Professor Qin Minjian, Department of Traditional Chinese Medicine Resources, China Pharmaceutical University. Ten compounds were isolated from the ethyl acetate extract of *Celastrus orbiculatus* (COE): diterpenoid lactones from *Prunus davidiana* A (I), (5β, 8α, 9β, 10α, 16β)-16-hydroxykaurane-18-oic acid (II), salicylic acid (III), 2,4,6–trimethoxyphenol-1-O-β-D- glucoside (IV), isoquercitrin (V), Quercetin-7-O- β-D-glucoside (VI), (+)-catechin (VII), vanillic acid (VIII), β-carotene (IX), β-sitosterol (X). The extraction and identification of these compounds were carried out by Professor Wang Qiang of China Pharmaceutical University. The detailed process of drug extraction was described previously (Zan et al., 2007; Li et al., 2012).

### Samples of Gastric Cancer

Gastric cancer specimens and corresponding adjacent non-tumor specimens were obtained from patients who underwent resectable therapy at The Affiliated Hospital of Yangzhou University, China. None of these patients received antitumor treatment before the operation, and the diagnosis of gastric cancer was pathologically confirmed. All samples were snap frozen in liquid nitrogen immediately after resection or biopsy and stored at −80°C until processing, This experiments were approved by the ethic committee of Medical College, YangZhou University, JiangSu, China.

### Cells and Reagents

Human gastric cancer cell line AGS was purchased from Shanghai cell bank, Institute of Cell Biology, Chinese Academy of Sciences (No. CRL-1739-LUC2). RPMI 1640 cell culture medium (GIBCO), fetal bovine serum (FBS) (Hyclone Laboratories Inc.) and trypsin were purchased from Shanghai Beyotime (China). MTT powder was purchased from Sigma-Aldrich. Matrigel and transwell chamber were purchased from BD Biosciences (United States). Monoclonal antibodies against Bcl-2, Bax, caspase-3 and PHB were purchased from Cell Signaling Technology (United States). β-actin antibody and HRP-labeled sheep anti rabbit IgG were purchased from Thermo Fisher Scientific (United States). BrdU staining kit was purchased from eBioscience Inc. (CA, United States). Annexin V-FITC kit was purchased from Miltenyi Biotec (Germany).

## Methods

### Cell Culture

AGS human gastric cancer cells were cultured in RPMI-1640 medium containing 10% FBS and incubated at 37°C in a humidity incubator containing 5% CO_2_.

### Clinical Samples of Gastric Cancer

#### Western Blot

AGS cells were inoculated in 6-well culture dishes and treated with RPMI 1640 medium containing COE in concentrations of 20, 40, and 80 mg/L for 24 h. Total protein was extracted from the treated cells in each group and then separated by SDS-PAGE. Then, the extracted protein was transferred to a PVDF membrane and the protein bands were detected by a gel imaging analysis system (Bio-Rad).

### Real Time Quantitative PCR (RT-qPCR)

AGS cells were inoculated in 6-well plates and incubated for 12 h. An equal amount of RPMI 1640 medium was added to the blank control group. To the COE group, RPMI 1640 medium was added containing 20, 40 and 80 mg/L COE in each well. After incubation for 24 h, the total RNA was extracted. The PHB and β-actin primers were synthesized by Shanghai Sangon Biotech Co., Ltd. (China). The PHB primers are is F5′-TGTCATTTTTTGACCGATTCCG-3′ and R5′-CTGGACATTACGTGGTCGAG-3′, and the amplified length was 125 bp. The β-actin primers are F5′- CAT​GTA​CGT​TGC​TAT​CCA​GGC-3′ and R5′- CTC​CTT​AAT​GTC​ACG​CAC​GAT-3′, and the amplified lenght was 250 bp. The average CT value, which is the number of cycles in which the fluorescence signal in each reaction tube reaches the defined value, was used to detect the target gene expression. The expression level of the control group was standardized to 1. The average of three experiments was taken for statistical analysis.

### MTT Assay

MTT assay was used to detect cell viability. Human gastric cancer AGS cells were evenly inoculated in 96-well culture plate, the cell number in each well was about 3,000–4,000. After overnight incubation, the cells were treated with different concentrations of COE (0, 10, 20, 40, 80, 160, and 320 mg/L) for 12, 24, and 48 h. Then, 20 μL of 5% MTT were added to each well and incubated at 37°C for 4 h. Subsequently, the supernatant was discarded and 150 μL of DMSO was added to each well. The crystal was fully dissolved by shaking at medium speed for 10 min. The absorbance (A) at 490 nm was determined from each well. The inhibition rate (%) was calculated as follows: [1—(A of cells in the drug group/A of cells in the control group)]  ×  100%. In order to evaluate the COE effect on cell growth, 50% inhibitory concentration (IC50) was calculated.

### Flow Cytometry Analysis

Annexin V-FITC kit was used to detect apoptotic and dead cells. Annexin V-FITC combined with phosphatidylserine (PS) positive cells were defined as apoptotic cells, while propidium iodide (PI) positive cells were defined as non-apoptotic cells. In brief, 10^6^ cells were resuspended in 100 μL 1 × binding buffer. Then, 10 μL Annexin V-FITC was added to each tube containing 10^6^ cells and the mixture was incubated in darkness at room temperature for 15 min. Then PI was added to the mixture immediately before low cytometry analysis and the sample was assessed on the computer.

### JC-1 Fluorescent Staining

JC-1 was used as fluorescent probe to detect the mitochondrial membrane potential. The change in the mitochondrial membrane potential is sensitive and needs rapid detection, which is often used to detect early apoptosis. Briefly, AGS cells were incubated with 1 ml of JC-1 working solution at 37°C for 20 min, washed three times with JC-1 buffer, and then analyzed by a confocal laser scanning microscope.

### Lentiviral Transfection

Human gastric cancer cells in the logarithmic growth phase were inoculated in a 6-well plate overnight. Then, the lentivirus (vectors: piLenti-siRNA-RFP, the details are shown in the table below) and the empty vector lentivirus were added to the culture medium separately and incubated for 24 h. Subsequently, transfection efficiency was observed under a fluorescence microscope. Western blot and PCR were used to verify the PHB inhibitory effect.

**Table:** 

NO.	Accession	Target Seq	Cds	Gc%
PHB-RNAi (57,877–1)	NM_001281496	TGC​CGT​CCA​TCA​CAA​CTG​A	147..965	52.63%
PHB-RNAi (57,878–1)	NM_001281496	GTG​GGT​ACA​GAA​ACC​AAT​T	147..965	42.11%
PHB-RNAi (57,879–1)	NM_001281496	TGT​TTG​AGT​CCA​TTG​GCA​A	147..965	42.1%
Description	Homo sapiens prohibitin (PHB), transcript variant 1, mRNA.			

### Subcutaneous Tumor Transplantation in Nude Mice

The 4 week-old nude mice (BALB/c-Nude) were purchased from the Comparative Medical Center of Yangzhou University. They were raised normally unyil 5 weeks of age. All animal experiments were carried out according to the “Laboratory animal care and use guide” of National Institutes of Health (NIH). Tumor cells in logarithmic growth phase were prepared in a 1 × 10^7^/ ml cell suspension with normal saline. Under sterile conditions, the skin was picked up at 15° angle and 10^6^ cells was injected subcutaneously into the right anterior armpit of mice until the hump was raised. The needle was removed quickly to prevent the cell suspension from overflowing the orifice. Tumor formation was observed every 7 days. This experiments were approved by the ethic committee of Medical College, YangZhou University, JiangSu, China.

### Fluorescence Technology of Small Animals *in vivo*

To study the COE effect on tumor cells *in vivo*, animal imaging technology was used. Human AGS cells transfected with red fluorescent protein (RFP). Tumor formation was observed after 7 days. After 14 days of the tumor cells having been inoculated, COE was injected intraperitoneally into nude mice with a tumor at a dose of 40 mg/kg. The fluorescence signals reflecting the tumor size of the mice were detected and analyzed by Perkin Elmer system to determine the tumor size.

### Statistical Analysis

SPSS 16.0 software was used for statistical analysis. The comparison between groups was performed by one-way ANOVA. The measurement data were expressed by mean ± standard deviation. The differences were statistically significant when *p < 0.05, **p < 0.01, ***p < 0.001.

## Results

**FIGURE 1:**
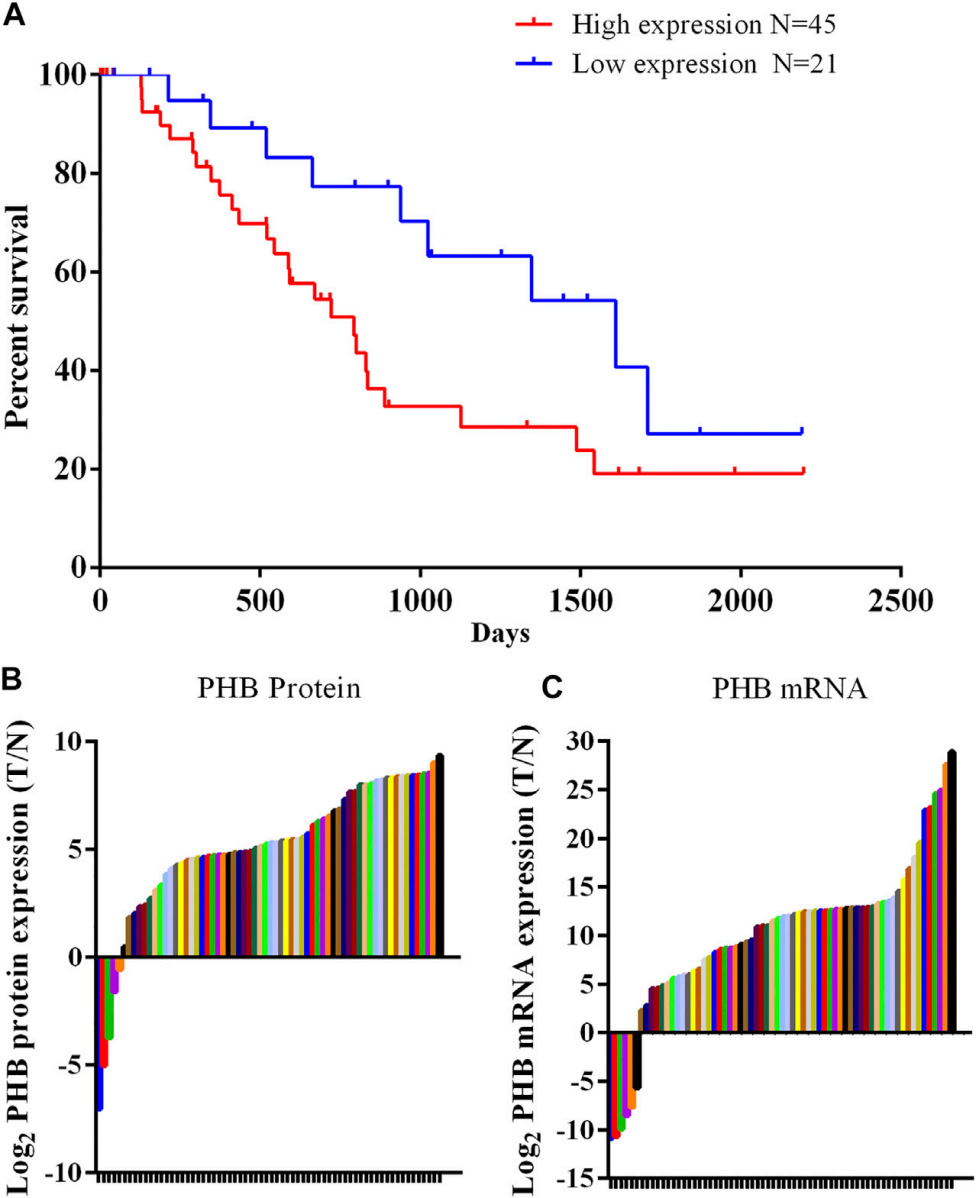
PHB expression in clinical samples and the relationship between PHB expression and the patient survival rate. **(A)** Patients with low PHB expression level present a higher survival rate those with high PHB expression level; **(B)** In 66 pairs of clinical samples, PHB protein expression in 61 tumor samples was higher than in adjacent tissues; **(C)** In 66 pairs of clinical samples, PHB mRNA expression in 60 tumor samples was higher than in adjacent tissues.

Western blot analysis of clinical samples showed that the expression of PHB1 protein in gastric cancer tissues was significantly higher than that in adjacent tissues (*p* < 0.05). The PCR results showed that PHB mRNA level in gastric cancer samples was significantly higher than in adjacent tissues (*p* < 0.05). According to the protein detection results and the survival information of patients, it was found that patients with low expression (Less than 3 times the average expression of adjacent tissues) of PHB had a statistically significant higher survival rate than those with high expression of PHB (Figure 1).

MTT assay was used to detect the COE effect on the proliferation and cell viability of AGS cells. The results showed that COE significantly inhibited the AGS cell proliferation with a certain dose dependence. As the COE concentration increases, AGS cell number decreases. Linear fit the inhibition rate and concentration through the ICsubscript calculator software, and calculate the equation. The 50% inhibitory concentration (ICsubscript) of COE on AGS cells was 68.24 mg / L (Table 1).

**TABLE 1:** The effect of COE on the proliferation of gastric cancer AGS cells (*‾x* ± *s*, *n* = 5).

Group (unit: mg/L)	Proliferation inhibition rate (%)
Control group (COE 0)	0 ± 0.354
COE 10	4.452 ± 0.877
COE 20	14.642 ± 1.212*
COE 40	30.255 ± 2.198**
COE 80	59.591 ± 4.013***
COE 160	80.845 ± 3.950***
COE 320	89.368 ± 1.034***

Note: Compared with Control Group, *P< 0.05, **P< 0.01, ***P< 0.001.

**FIGURE 2:**
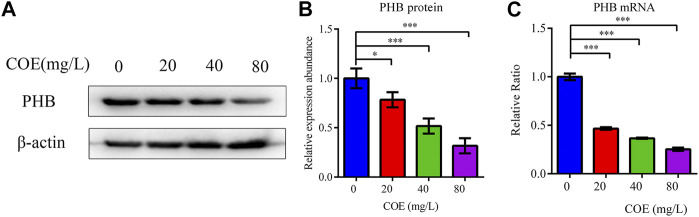
Effect of the ethyl acetate extract of *Celastrus orbiculatus* (COE) on PHB mRNA **(A)** and protein expression **(B)**.

In order to study the effect of COE on the mRNA and PHB protein expression, the total protein and RNA of AGS cells were extracted after treatment with COE for 24 h. RT-qPCR and Western blot were used to detect PHB expression and they showed that the PHB expression at mRNA and protein level were significantly inhibited by COE (Figure 2), (*n* = 5).

**FIGURE 3:**
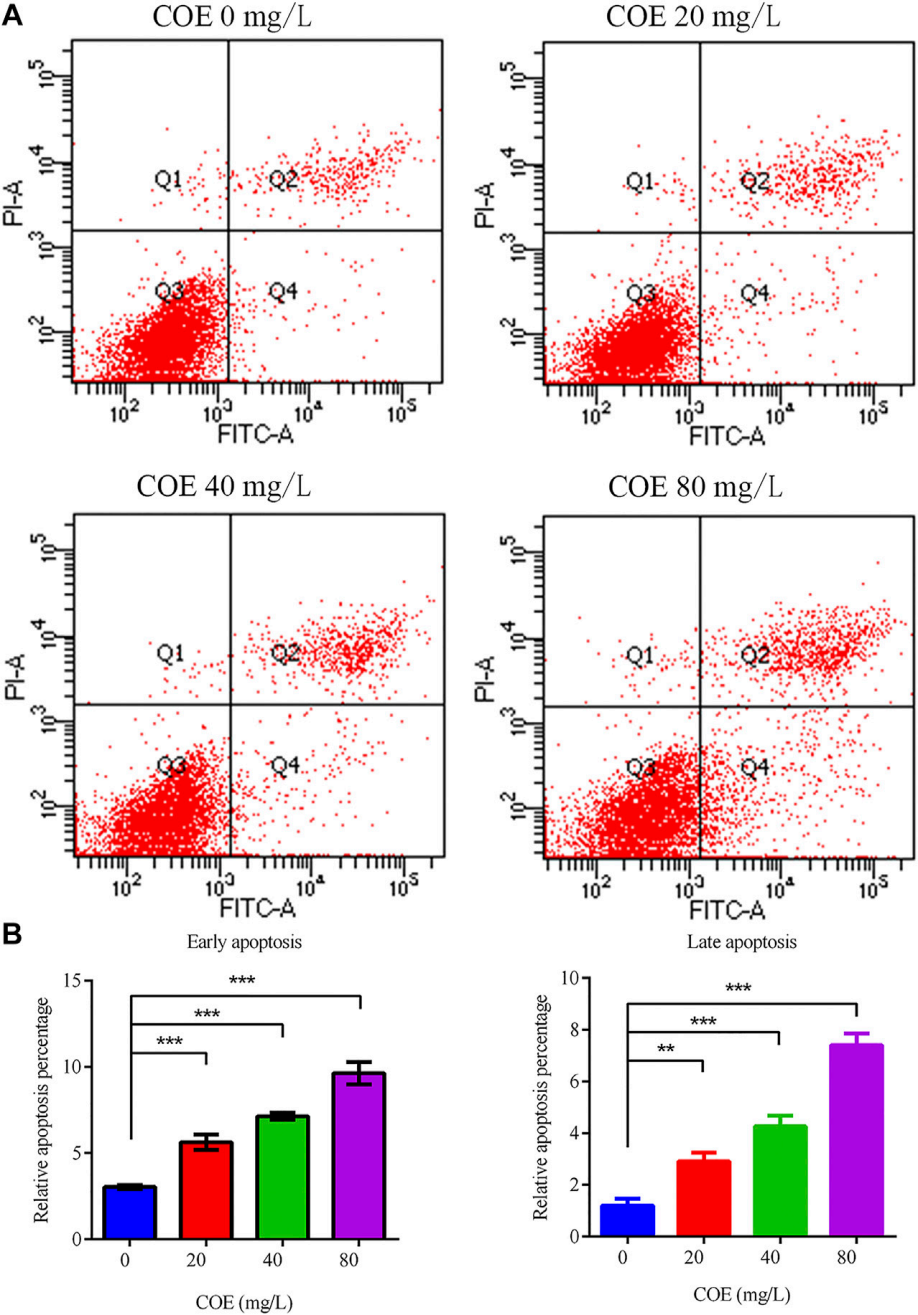
Effect of the ethyl acetate extract of *Celastrus orbiculatus* (COE) on early and late apoptosis of human gastric cancer AGS cells. **(A)** and **(B)** COE can significantly promote the apoptosis of AGS cells.

Annexin V-FITC/PI dual staining was performed on AGS cells treated with COE and analyzed by flow cytometry to study the COE inhibition mechanism on the proliferation of these cells. The results showed that COE can significantly promote AGS cell apoptosis compared to the control group (*p* < 0.05). As COE concentration increased, the apoptotic cell number also increased (Figure 3), (*n* = 5).

**FIGURE 4:**
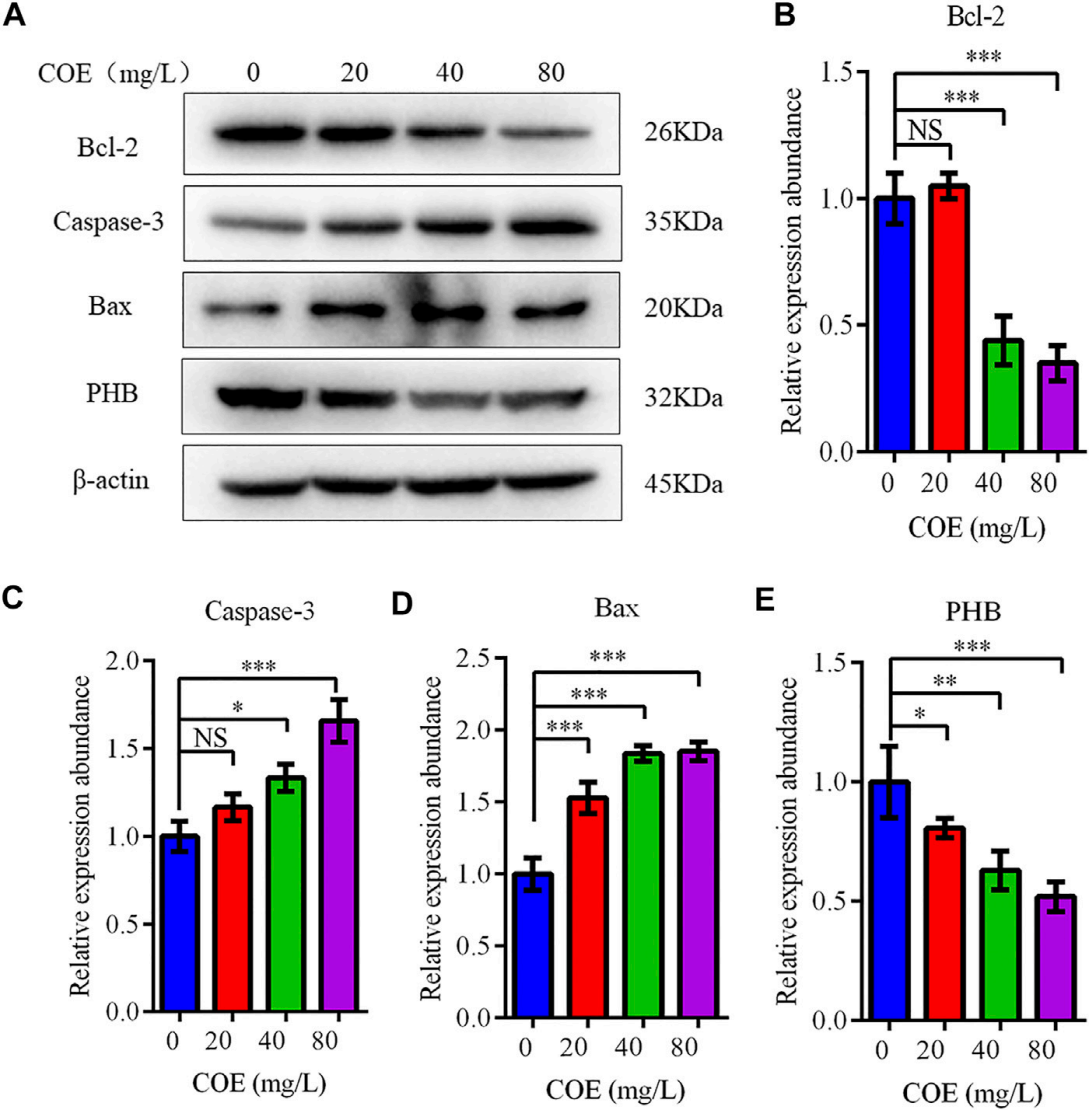
Effect of the ethyl acetate extract of *Celastrus orbiculatus* (COE) on expression of apoptosis-related proteins in human gastric cancer AGS cells. Bcl-2 expression decreased significantly, while Bax and Caspase-3 expression increased significantly after COE treatment **(A–E)**.

The expression level of Bcl-2, Bax and caspase-3 apoptosis-related proteins was measured to confirm COE apoptosis-promoting effect on AGS cells. In fact, Western blot results showed that Bcl-2 expression decreased significantly, while the Bax and caspase-3 expression increased significantly after COE treatment (Figure 4; *p* < 0.05), (*n* = 5).

**FIGURE 5:**
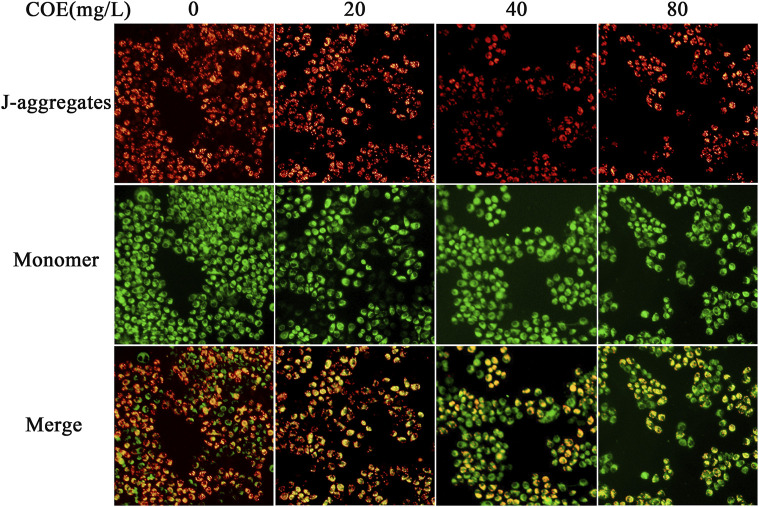
Effect of the ethyl acetate extract of *Celastrus orbiculatus* (COE) on mitochondrial membrane potential of human gastric cancer AGS cells. The mitochondrial membrane potential of AGS cells changed significantly after COE treament.

In order to reveal the pathway by which COE promotes apoptosis of AGS cells, JC-1 mitochondrial membrane potential assay was used to detect the COE effect on mitochondrial function of these cells. The results showed that the mitochondrial membrane potential of AGS cells changed significantly after 24 h treatment with COE (Figure 5; *p* < 0.05).

**FIGURE 6:**
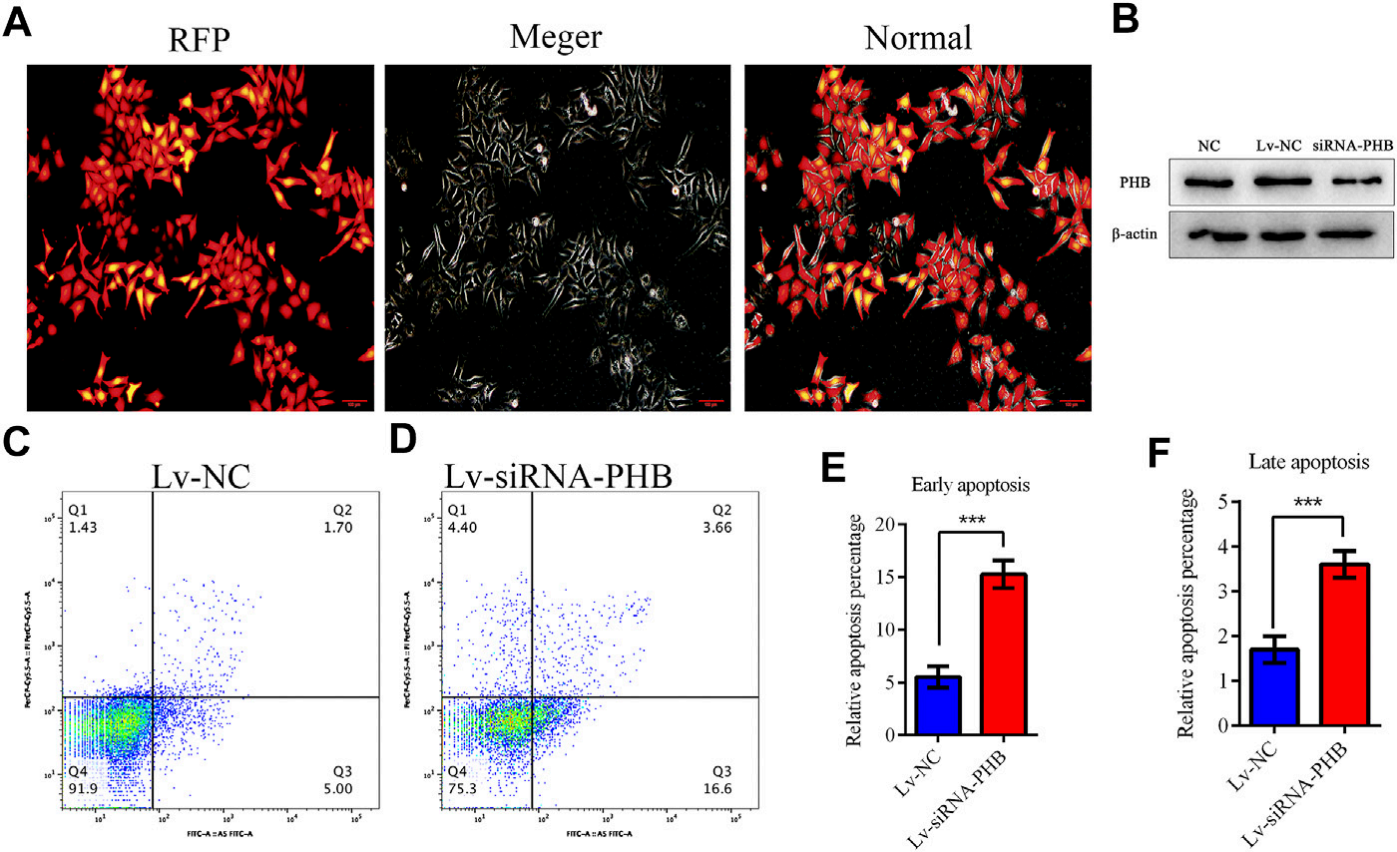
PHB plays a key role in inducing human gastric cancer AGS cell apoptosis. **(A)** and **(B)** Lentivirus-mediated interference RNA can knockdown the PHB expression in AGS cells. **(C–F)** PHB expression knockdown increased significantly, the apoptosis rate of AGS cells, indicating that PHB deletion can promote the apoptosis of these cells.

PHB knockdown was induced by transfecting lentivirus-mediated interfering RNA into AGS cells to verify whether COE can change the PHB role in mitochondrial function by inhibiting PHB expression and thus activating mitochondrial apoptosis pathway. The results showed that PHB knockdown significantly increased the apoptosis rate of AGS cells (Figure 6; *p* < 0.05), suggesting that the PHB deletion may promote the AGS cell apoptosis.

**FIGURE 7:**
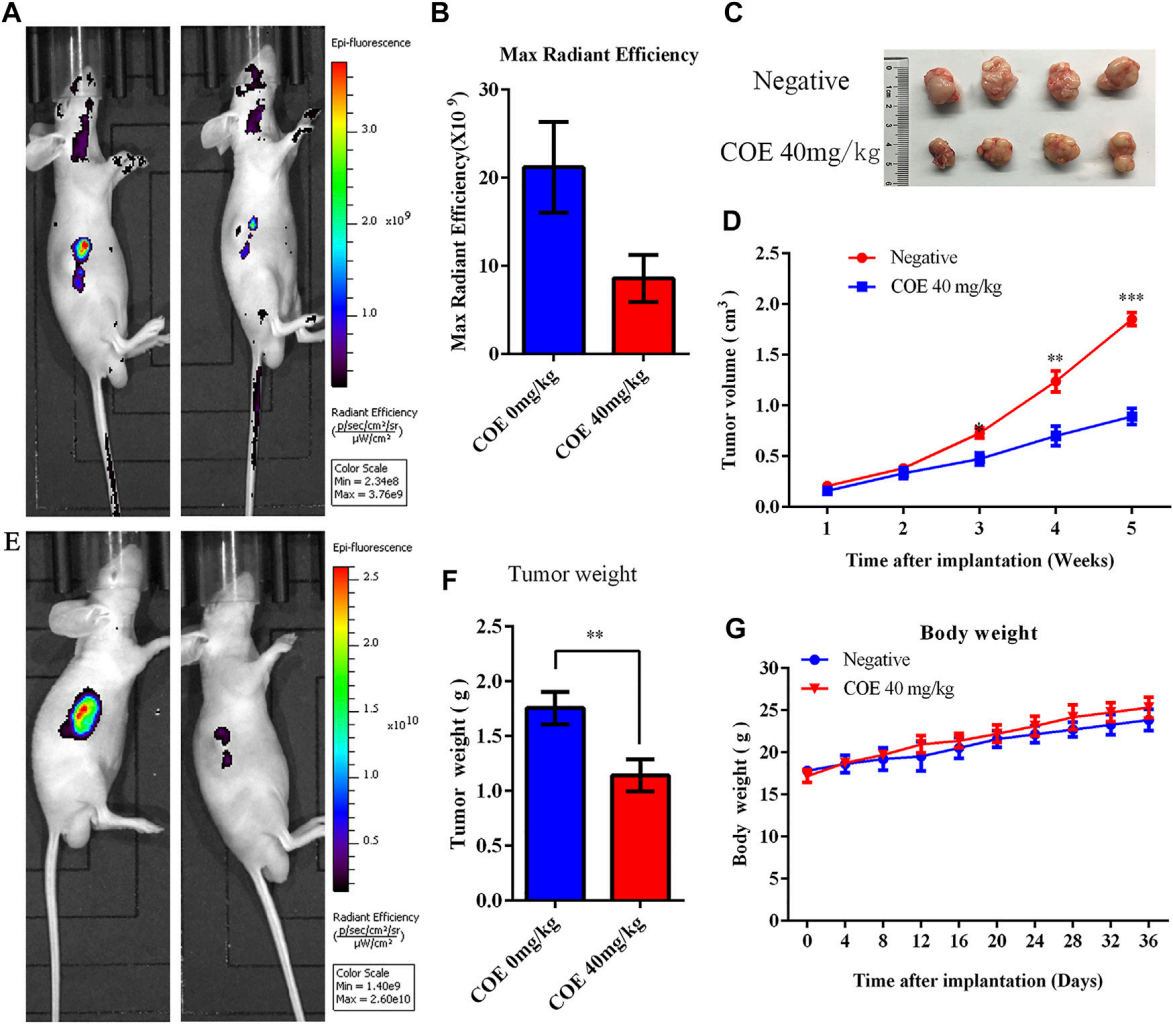
Ethyl acetate extract of *Celastrus orbiculatus* (COE) inhibited the growth of gastric tumor xenografts in nude mice *in vivo*. **(A)** and **(E)** *In vivo* imaging of small animals have shown that COE can inhibit gastric tumor growth *in vivo*. **(B)** Statistical results of the imaging fluorescence intensity. **(C)** and **(F)** Size and weight of the transplanted tumors. **(D)** Volume growth curve of the tumor. **(G)** Weight growth curve of nude mice.

Xenograft tumor experiments in nude mice were conducted to verify the COE apoptosis-promoting effect on gastric cancer cells *in vivo*. Mice were given 40 mg/kg of COE by oral gavage for 21 days. The mice body weight was measured every three days and the tumor volume was measured once a week. The tumor size of nude mice was detected by fluorescence *in vivo* before and after oral gavage. The results showed that the growth of the subcutaneous transplanted tumor in nude mice was significantly inhibited after 21 days of COE intragastric administration (*p* < 0.05). In addition, it was found that the tumor weight in the COE treatment group was significantly smaller than in the control group (Figure 7; *p* < 0.05).

## Discussion

Traditional Chinese medicine has become a hot spot in the research of antitumor drugs and the source of candidate materials (Huang et al., 2013; Bai et al., 2014; Yu et al., 2015). In this study, we investigated the COE effect on gastric cancer and analyzed the molecular mechanism of COE-induced programmed cell death (Wang et al., 2017). Our results lay the foundation for the development of new and effective anticancer drugs. In this study, we found that PHB is differentially expressed in gastric cancer and adjacent tissues. PHB is one of the important regulators of cell mitochondrial function stability (Merkwirth and Langer, 2009; Mishra et al., 2010; Signorile et al., 2019 Jan), The function and structural stability of mitochondria directly affect the energy supply and apoptosis of tumor cells. Through research, we found that COE can significantly promote the early and late apoptosis of AGS cells. In order to further clarify the mechanism of COE on AGS cell viability and apoptosis, a series of biological experiments were performed here. Quantitative analysis results show that the anti-tumor effect of COE is mainly achieved by promoting tumor cell apoptosis. As an important regulator of mitochondrial function stability (Signorile et al., 2019 Jan), the role of PHB in COE’s promotion of gastric cancer cell apoptosis has gradually been revealed by us. COE has been found to significantly reduce mRNA and PHB protein expression in gastric cancer cells, and that the decrease in PHB expression directly affects mitochondrial function. The structural and functional stability of mitochondria can directly affect cell energy metabolism and the triggering of apoptosis mechanisms (Mishra et al., 2010; Chowdhury et al., 2014). Therefore, we hypothesized that the apoptosis of COE-induced apoptosis may occur due to the inhibition of PHB expression and the destruction of mitochondrial function, thus activating the mitochondrial apoptosis pathway and resulting in cell apoptosis. Decreased mitochondrial membrane potential is a marker event in the early stage of apoptosis (Merkwirth and Langer, 2009; Zhang et al., 2012; Chowdhury et al., 2013; Peng et al., 2015; Hong et al., 2018). In the present work, the results showed that COE significantly changed the mitochondrial membrane permeability and decreased the mitochondrial membrane potential, which is consistent with our hypothesis.

To clarify the molecular mechanism by which COE promotes apoptosis, we also evaluated the expression of apoptosis-related proteins. Changes in the mitochondrial membrane potential are mainly regulated by Bcl-2 family proteins. The decrease in the Bcl-2 level leads to changes in mitochondrial membrane permeability and the cytochrome c release, which allows the formation of apoptotic complexes and activation of caspase family proteins, including Caspase-3 (Xu et al., 2016; Cho et al., 2019; Ortiz et al., 2019). Caspase-3 activation triggers the final process of apoptosis (Zhang et al., 2012). Taken together, these data indicate that COE activates the mitochondria-mediated apoptosis pathway and caspase-3, which is consistent with the results of MTT, flow cytometry and mitochondrial membrane potential experiments.

The COE antitumor effect was further verified by *in vivo* xenograft tumor experiments. The results showed that COE significantly inhibited the gastric cancer tumor growth *in vivo*, which is in agreement with the *in vitro* experiments results. Similar to numerous studies, these data suggest that natural products are capable of inducing apoptosis through the mitochondrial pathway. Some studies have shown that the mitochondrial structure of gastric cancer cells has changed significantly after COE treatment (Wang et al., 2017). Mitochondria are the main source of energy for cells and mitochondrial dysfunction directly affects the normal biological behavior of cells, including cell division and proliferation. Incomplete mitochondrial membrane structure also accelerated the release of cytochrome c and promoted apoptosis (Sripathi et al., 2016; Djehal et al., 2018; Jang et al., 2018; Wang et al., 2020).

In conclusion, COE can significantly induce gastric cancer cell apoptosis *in vivo* and *in vitro* by inhibiting the expression of PHB protein, destroying the stability of mitochondrial function and activating mitochondrial apoptosis pathway. Therefore, COE is a mixture natural compounds with a notorius ant-tumor effect, with great potential application in the gastric cancer treatment.

## Data Availability

The original contributions presented in the study are included in the article/Supplementary Material, further inquiries can be directed to the corresponding authors.
