# Corrigendum: The Ethyl Acetate Extract From Celastrus Orbiculatus Promotes Apoptosis of Gastric Cancer Cells Through Mitochondria Regulation by PHB

**DOI:** 10.3389/fphar.2022.835426

**Published:** 2022-03-11

**Authors:** Lide Tao, Zixin Yin, Tengyang Ni, Zewen Chu, Shihua Hao, Zeyu Wang, Masataka Sunagawa, Haibo Wang, Yanqing Liu

**Affiliations:** ^1^ Nanjing University of Traditional Chinese Medicine, Nanjing, China; ^2^ Department of General Surgery, Affiliated Hospital of Yangzhou University, Yangzhou University, Yangzhou, China; ^3^ Institute of Translational Medicine, Medical College, Yangzhou University, Yangzhou, China; ^4^ Dalian Medical University, Dalian, China; ^5^ Department of Physiology, School of Medicine, Showa University, Tokyo, Japan

**Keywords:** celastrus orbiculatus extract, prohibitin, gastric cancer, traditional Chinese medicine, apoptosis

In the original article, there was a mistake in Figure 5 as published. We performed the same experiment in different cell lines and at the same time, their experimental results were consistent. Consequently, due to our negligence, we accidentally used pictures of other cell lines in the picture when drawing the picture. The BGC-823 cell line was mislabeled as the AGS cell line. The corrected Figure 5 appears below.

**FIGURE 5:**
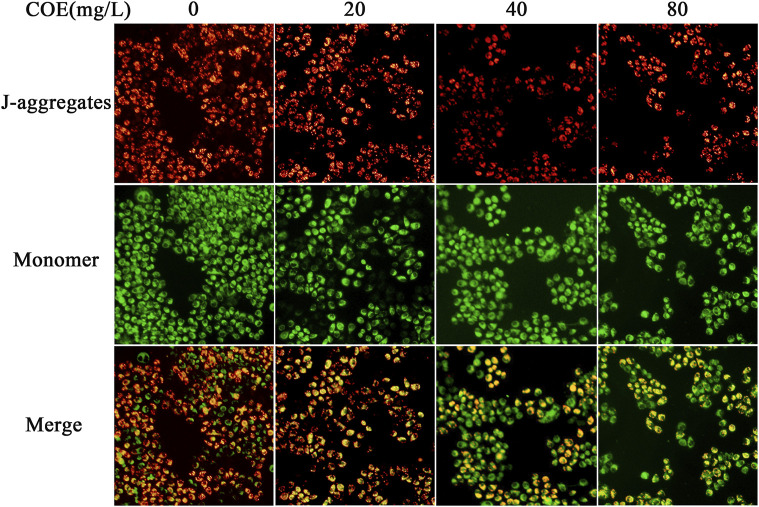
Effect of the ethyl acetate extract of Celastrus orbiculatus (COE) on mitochondrial membrane potential of human gastric cancer AGS cells. The mitochondrial membrane potential of AGS cells changed significantly after COE treament.

The authors apologize for this error and state that this does not change the scientific conclusions of the article in any way. The original article has been updated.

