# Supplementary figures and images for: The Ethyl Acetate Extract From Celastrus orbiculatus Promotes Apoptosis of Gastric Cancer Cells Through Mitochondria Regulation by PHB
Source: Front Pharmacol. 2021 May 28;12:635467. doi: 10.3389/fphar.2021.635467 (PMC8194300; doi:10.3389/fphar.2021.635467)

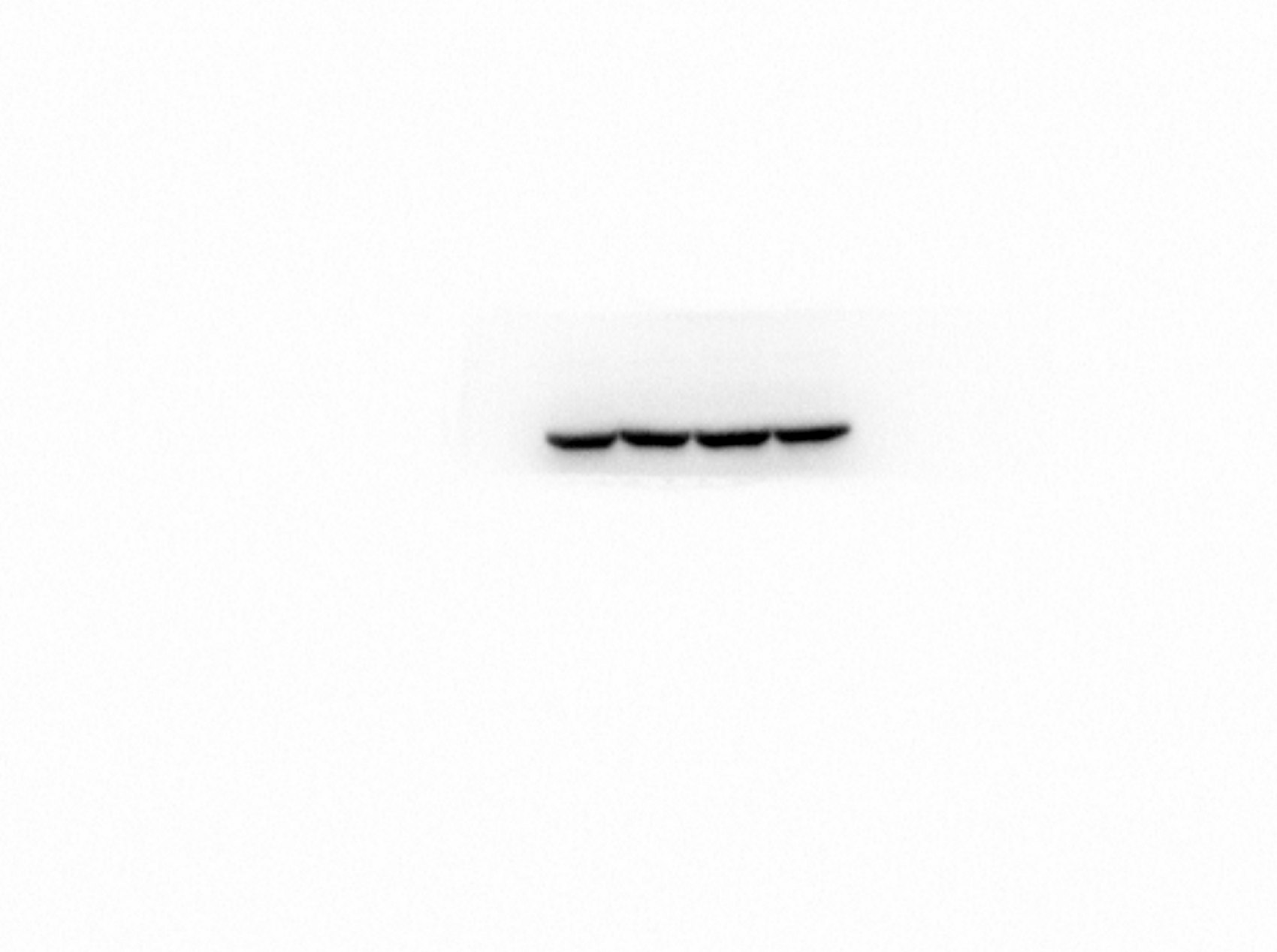

Supplement: Supplementary file 1 [file DataSheet1.zip › 新建文件夹/actin dw jianqiehou.tif]

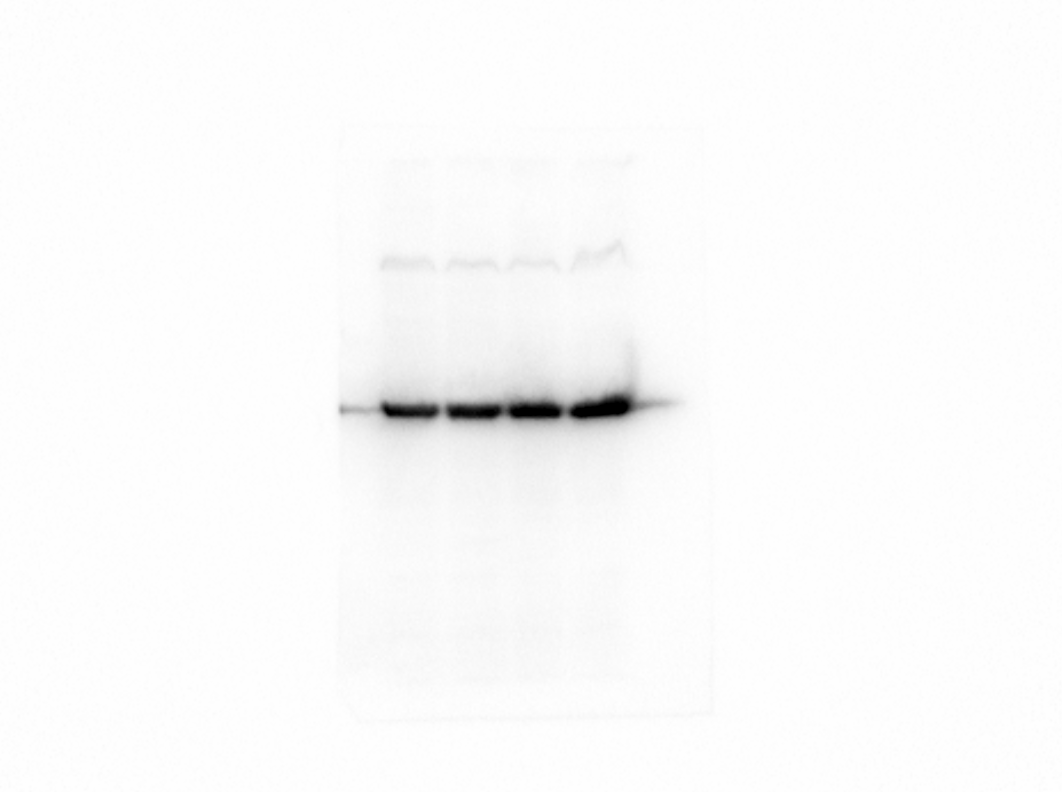

Supplement: Supplementary file 1 [file DataSheet1.zip › 新建文件夹/b-actin.tif]

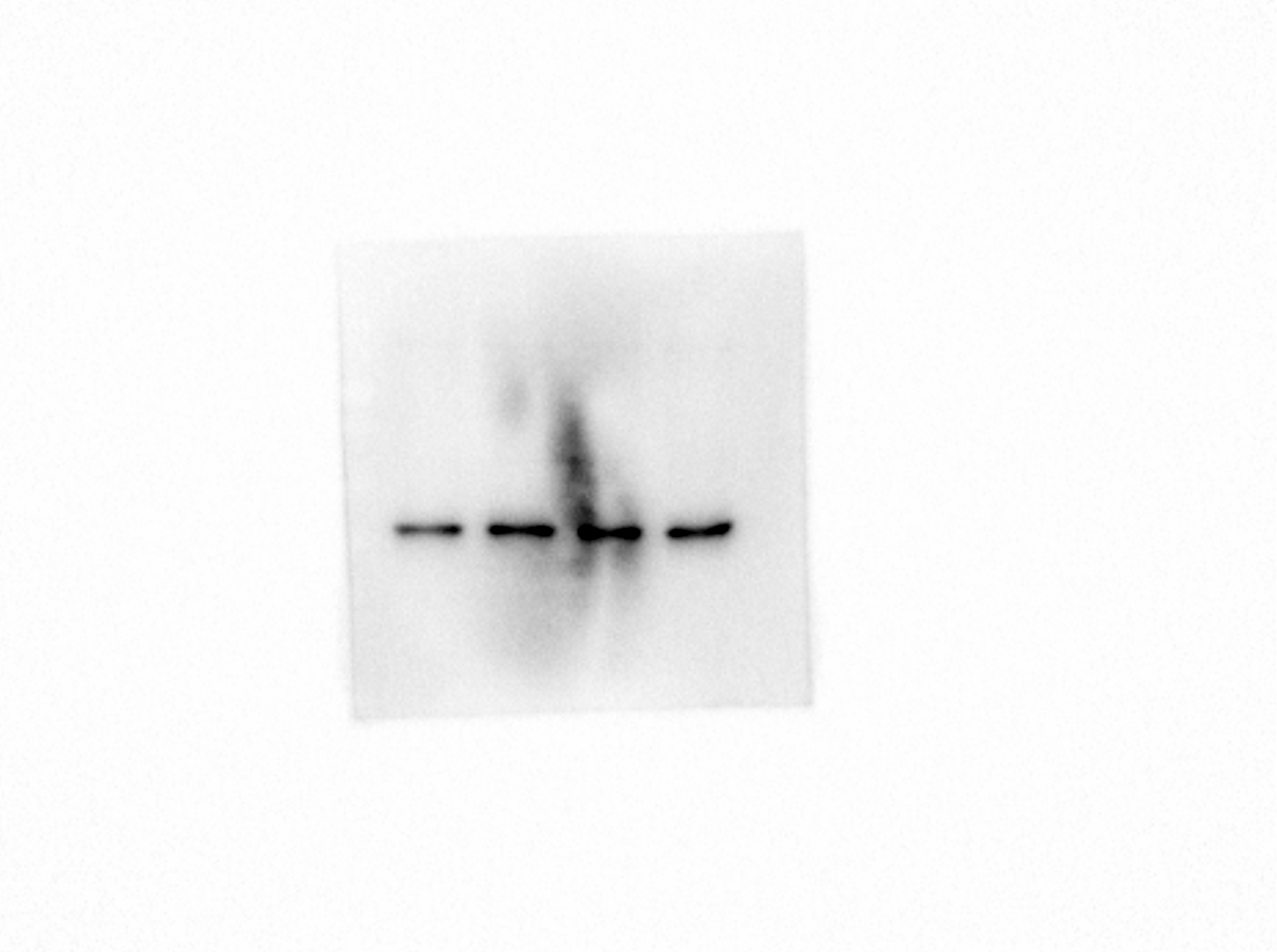

Supplement: Supplementary file 1 [file DataSheet1.zip › 新建文件夹/BAX.jpg]

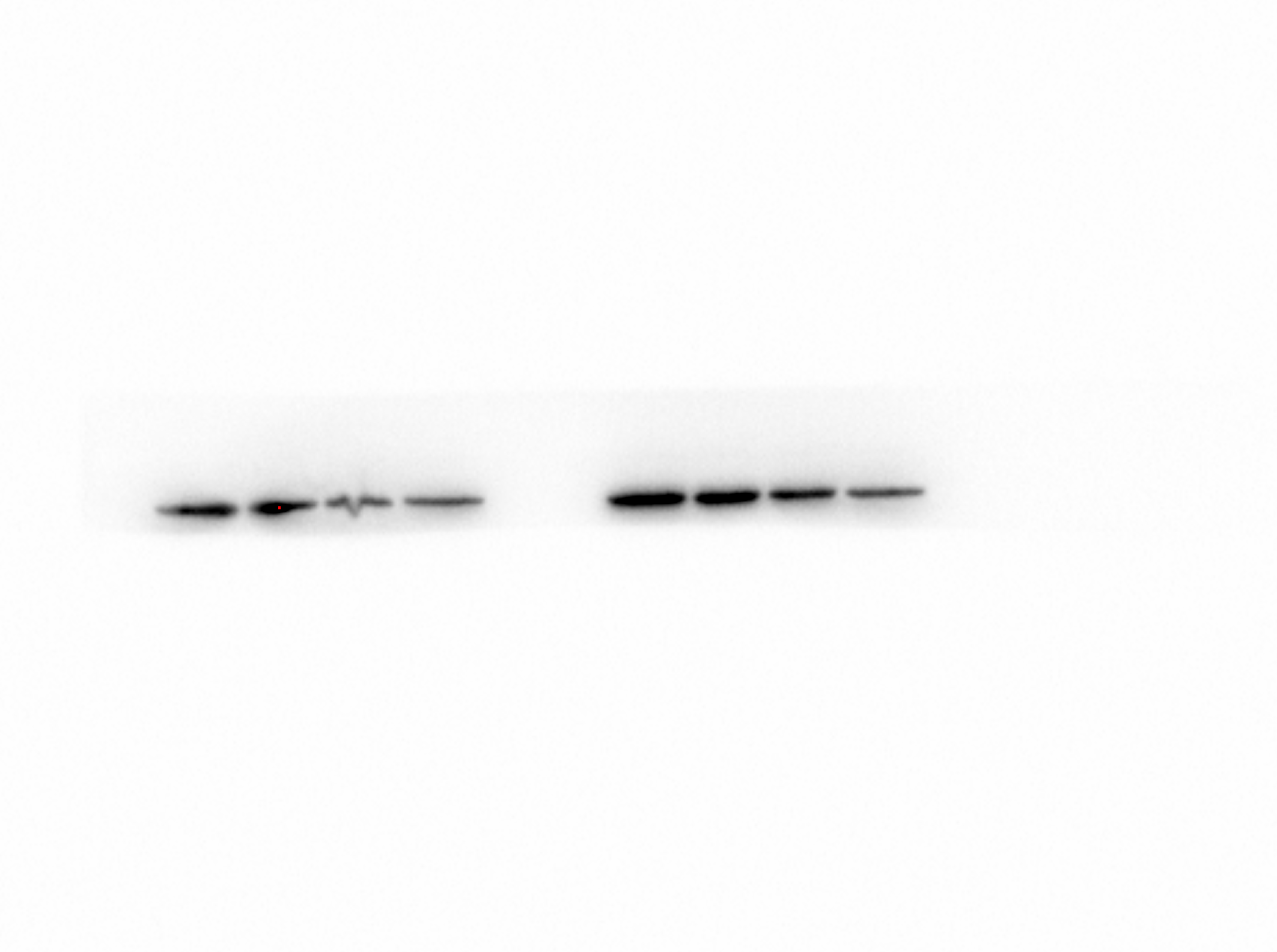

Supplement: Supplementary file 1 [file DataSheet1.zip › 新建文件夹/bcl-2 (3).tif]

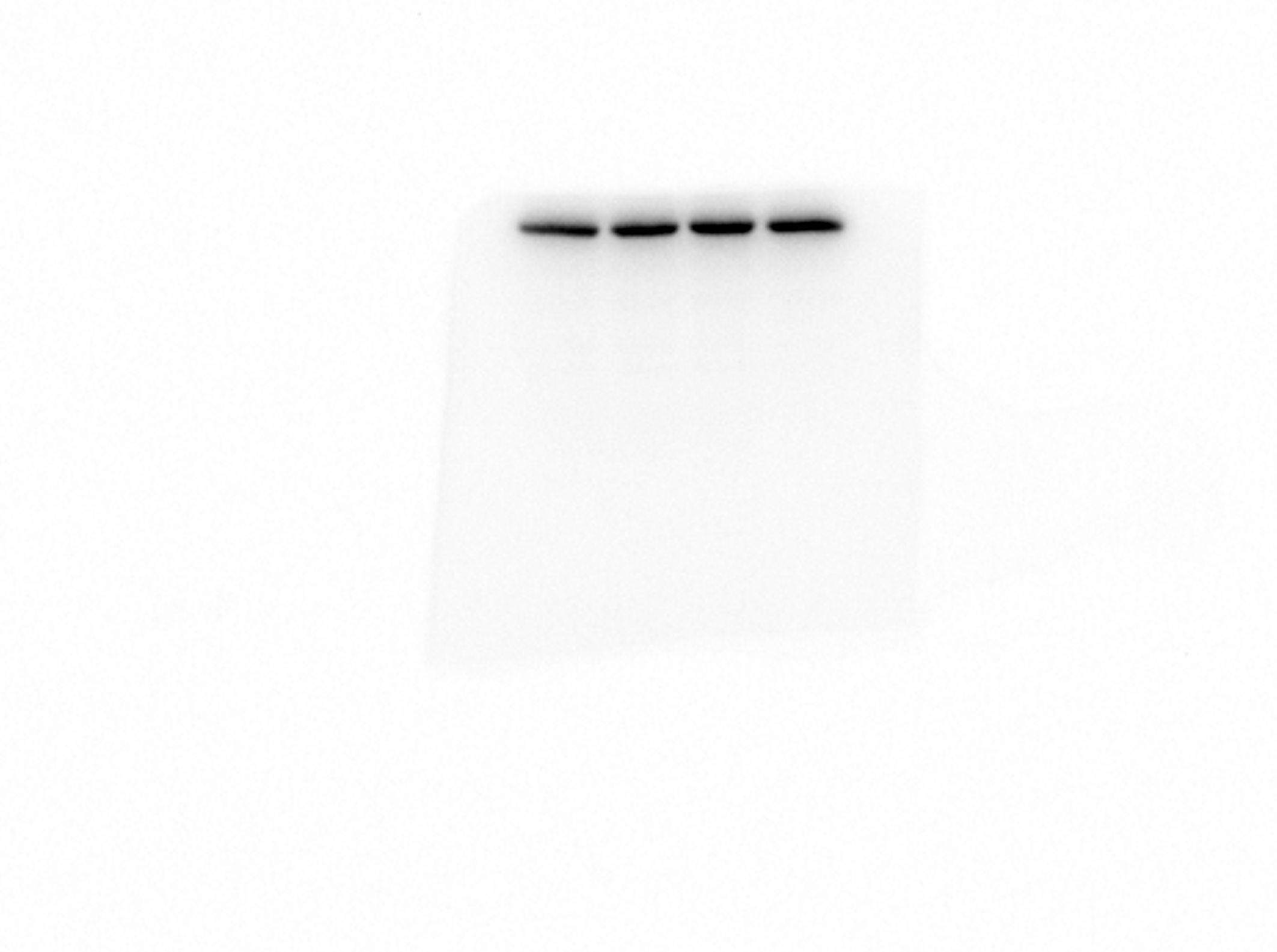

Supplement: Supplementary file 1 [file DataSheet1.zip › 新建文件夹/caspase1.tif]

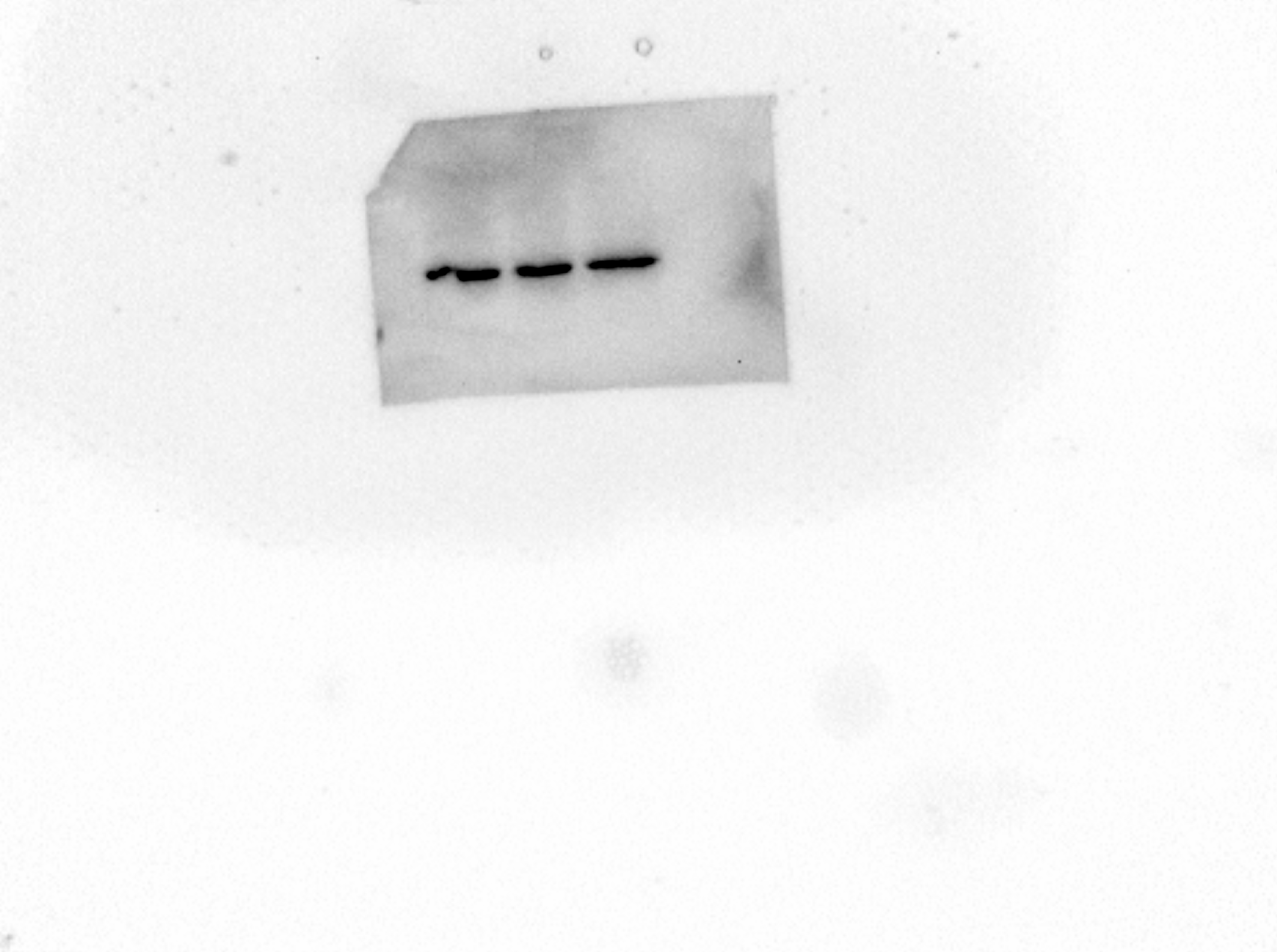

Supplement: Supplementary file 1 [file DataSheet1.zip › 新建文件夹/duan2 ACTIN.tif]

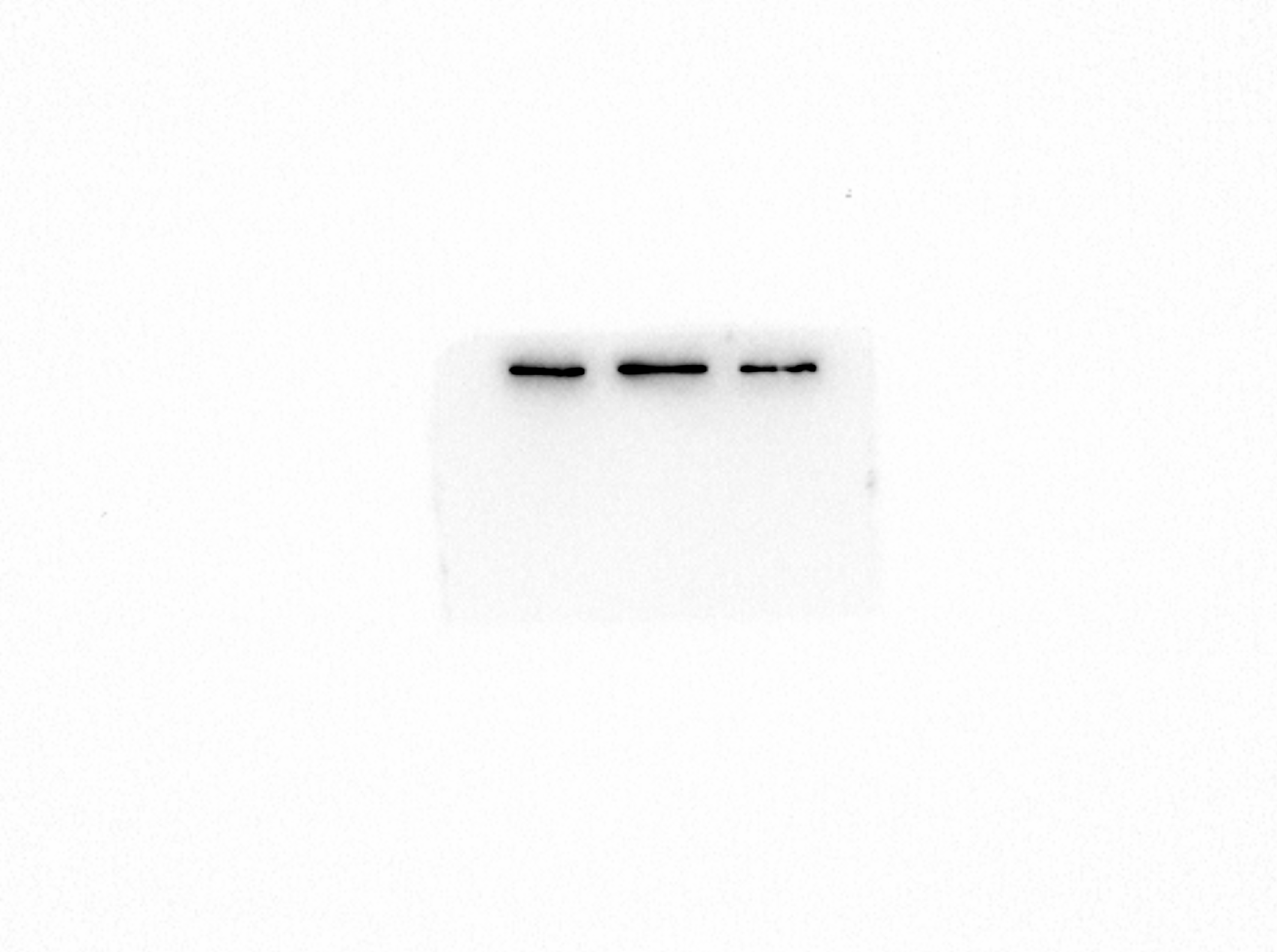

Supplement: Supplementary file 1 [file DataSheet1.zip › 新建文件夹/k,v,p actin .tif]

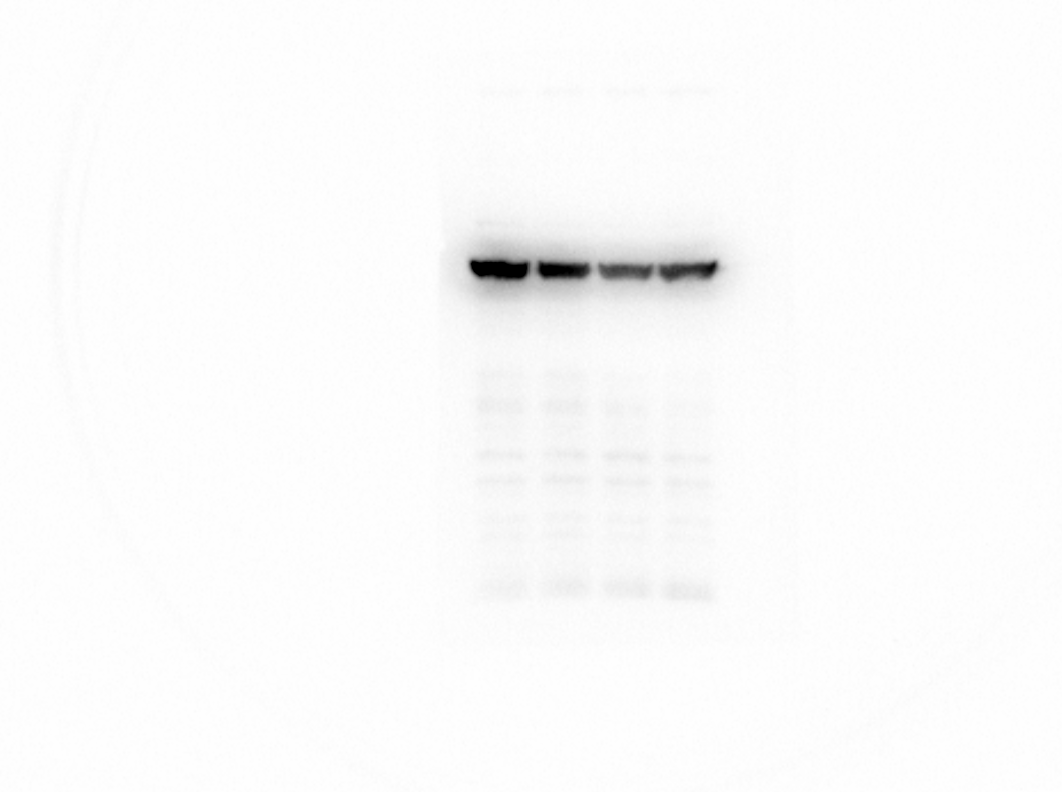

Supplement: Supplementary file 1 [file DataSheet1.zip › 新建文件夹/phb (2).tif]

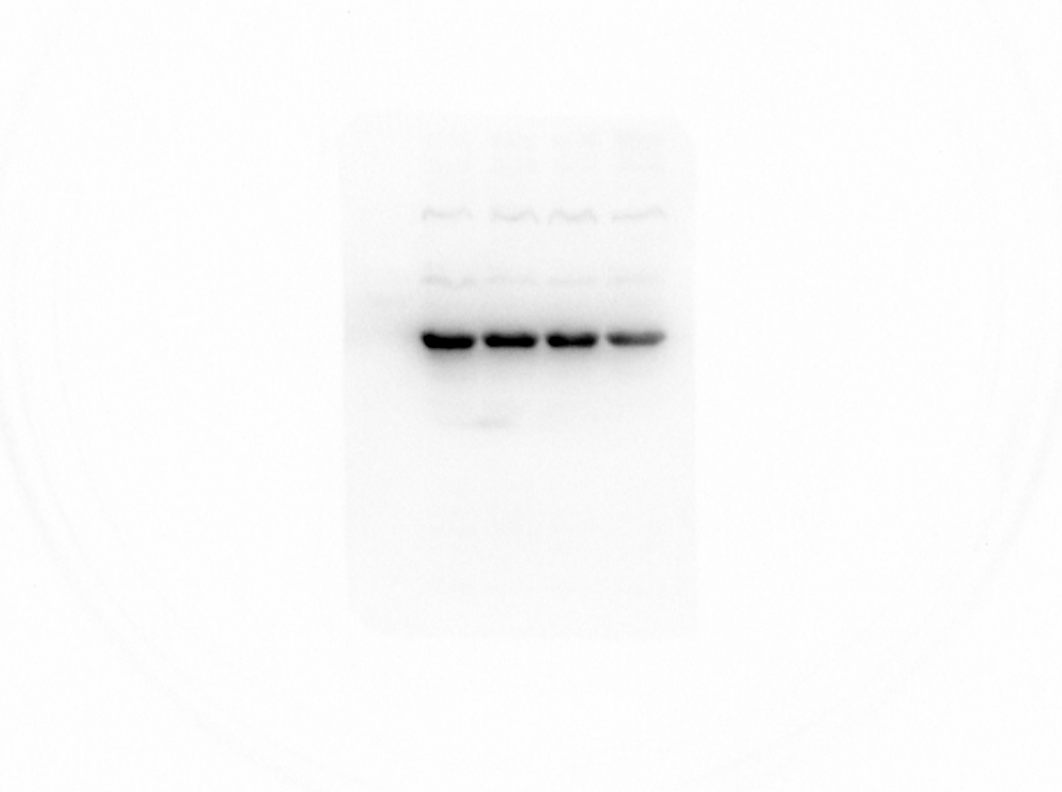

Supplement: Supplementary file 1 [file DataSheet1.zip › 新建文件夹/phb1.tif]
